# The Relative Contributions of Visual and Proprioceptive Inputs on Hand Localization in Early Childhood

**DOI:** 10.3389/fnhum.2021.702519

**Published:** 2021-10-07

**Authors:** Natasha Ratcliffe, Katie Greenfield, Danielle Ropar, Ellen M. Howard, Roger Newport

**Affiliations:** ^1^School of Psychology, University of Nottingham, Nottingham, United Kingdom; ^2^School of Psychology, University of Southampton, Southampton, United Kingdom; ^3^School of Sport, Exercise and Health Sciences, Loughborough University, Loughborough, United Kingdom

**Keywords:** multisensory integration, sensory processing, vision, proprioception, development

## Abstract

Forming an accurate representation of the body relies on the integration of information from multiple sensory inputs. Both vision and proprioception are important for body localization. Whilst adults have been shown to integrate these sources in an optimal fashion, few studies have investigated how children integrate visual and proprioceptive information when localizing the body. The current study used a mediated reality device called MIRAGE to explore how the brain weighs visual and proprioceptive information in a hand localization task across early childhood. Sixty-four children aged 4–11 years estimated the position of their index finger after viewing congruent or incongruent visuo-proprioceptive information regarding hand position. A developmental trajectory analysis was carried out to explore the effect of age on condition. An age effect was only found in the incongruent condition which resulted in greater mislocalization of the hand toward the visual representation as age increased. Estimates by younger children were closer to the true location of the hand compared to those by older children indicating less weighting of visual information. Regression analyses showed localizations errors in the incongruent seen condition could not be explained by proprioceptive accuracy or by general attention or social differences. This suggests that the way in which visual and proprioceptive information are integrated optimizes throughout development, with the bias toward visual information increasing with age.

## Introduction

The ability to locate our body parts in space is fundamental for successful interaction with the environment and plays a vital role in developing a sense of the bodily self. In order to understand and interact with the environment around the body, the brain must integrate information from multiple sensory modalities to construct unified representations of the bodily self and the world around it. The integration of proprioceptive, somatosensory and visual inputs specifically underpins the subjective sense of self and body ownership (Makin et al., 2008), which in turn are important for the development of self-awareness and social cognition (Schütz-Bosbach et al., 2006).

How the brain integrates sensory information in order to make sense of the body has been studied extensively in adulthood. Studies (e.g., Alais and Burr, 2004; Trommershauser et al., 2011) show that the degree to which adults integrate sensory inputs can be quantitatively predicted by a Maximum-Likelihood-Estimate (MLE) model of optimal integration (van Beers et al., 1996; Ernst and Banks, 2002). For example, when judging the size of an object, estimates of size derived from each sense are averaged and combined to construct a coherent percept. These estimates are prone to variance but, by averaging the estimates, the brain can reduce the noise in the overall percept (Landy et al., 1995). Specifically, a greater weighting will be given to estimates with less variance, since these are deemed as more reliable. The degree of variance in an estimate is dependent on both bottom-up processes (i.e., the incoming sensory information) and top-down processes (derived from prior knowledge and experience).

In support of this model, research finds that in adults no single sense totally dominates bodily experience; instead the experimental context and prior information predicts which sense is treated as more reliable and hence given a greater weighting (van Beers et al., 2002). For example, proprioceptive inputs are weighted more strongly when adult participants actively move the hand compared to when it is passively placed by another person (Mon-Williams et al., 1997) because active movement provides richer and more reliable sensory information about limb position. Similarly, while visual cues are relied on more than proprioceptive information when perceiving limb position (Hay et al., 2014), the reverse is found when visual information is limited to a small light attached to one finger (Plooy et al., 1998). In addition, simply looking toward an unseen hand can change the weighting of sensory information and improve proprioceptive localization (Newport et al., 2001). Together, these findings support the argument that adults integrate information from multiple modalities in a statistically optimal way by taking into account the precision of inputs in different circumstances (van Beers et al., 1999). However, it is not clear when this ability to optimally integrate visual, proprioceptive and tactile information underlying body representation develops in children.

Though studies in early to late childhood have been conducted, a review on the development of multisensory integration abilities concluded that the age at which optimal integration occurs is still unclear (Dionne-Dostie et al., 2015). Charting the development of visuo-tactile-proprioceptive integration in children is important because it has been suggested that typical integration is necessary for higher order processes such as body ownership and social skills (Gallese, 2003; Gallese et al., 2004; Chaminade et al., 2005). A wide body of research has established that both a sense of self (Rochat, 2010; Lewis, 2011) and social processing skills (Merrell and Gimpel, 2014) develop and mature with age. Furthermore, research working with autistic children has indicated a relationship between atypical visuo-proprioceptive integration and the severity of social difficulties (Cascio et al., 2012). Investigating the integration of these inputs in typical development can increase our understanding of the mechanisms underlying the development of social behaviors and provide a comparison point to assess the nature of atypical multisensory integration in neurotypical conditions.

Based on adult research, a common method used to investigate how children combine multisensory information is by introducing conflict between cues from different senses. Research using preferential looking paradigms has demonstrated that infants even a few months old can detect temporal delays between visuo-tactile inputs (Zmyj et al., 2011; Filippetti et al., 2013, 2014; Freier et al., 2016) and visuo-proprioceptive information related to their bodies (Bahrick and Watson, 1985; Rochat and Morgan, 1995; Schmuckler, 1996; Morgan and Rochat, 1997). However, although these findings suggest that infants may be sensitive to visuo-tactile and visuo-proprioceptive contingencies, it cannot tell us if they actually derive a sense of bodily self or body ownership from this (Bremner et al., 2012). Moreover, preferential looking studies cannot assess the relative weighting given to different senses and thus whether infants integrate multisensory information in an optimal, adult-like manner. Research examining the development of postural control has shown that children as young as 4 years old are able to integrate sensorimotor signals and re-weight these in response to changing sensory environments; however, the magnitude of this re-weighting increases with age over childhood and does not become adult-like until around 12 years of age (Barela et al., 2003; Bair et al., 2007; Polastri and Barela, 2013).

Other studies which have also found evidence for a protracted period of development for sensory integration have employed the rubber hand illusion (RHI) (Cowie et al., 2013, 2016). In the RHI a fake hand is embodied following simultaneous felt and seen touch applied to an individual’s unseen hand and a fake hand, respectively. Estimates of body ownership of the fake hand are assessed through explicit questions of body ownership and through hand localization via pointing to the position of their unseen hand. In Cowie et al.’s (2013) study, when visual-tactile inputs were synchronous, both adults and children aged 4—9-years-old estimated the location of their unseen hand to be closer to the fake hand than in pre-touch baseline conditions—an indication that multisensory integration had taken place. However, unlike adults, even when visual-tactile inputs were asynchronous, 4–9 year old children’s made estimates were also closer to the fake hand than in baseline conditions which might suggest either that visual capture by the fake hand dominates proprioception or that the temporal binding of visuo-tactile sensory information is not as tightly constrained in younger children as it is in older children and adults (Greenfield et al., 2015, 2017). Therefore, the involvement of temporal processing in the RHI paradigm, makes it more difficult to determine the weighting of different sensory inputs.

Other research which has been able to more clearly assess the relative weighting of specific sensory inputs in early childhood have used hand localization tasks. King et al. (2010) used a sensory conflict paradigm to assess visuo-proprioceptive integration in 7-13-year-olds. Children pointed to a visual or a proprioceptive target (the unseen finger of their other hand), with or without the addition of a visual marker (i.e., circle), which was either congruent or incongruent with the location of the unseen finger. When congruent visual and proprioceptive information was available, children’s estimates were more reliable than in conditions when information from only one modality is present. This indicates that 7–13-year-olds are able to flexibly re-weight sensory information according to the task demands. However, in an incongruent condition in which the visual marker and proprioceptive target (the unseen finger) were in conflicting locations, older children increased the weighting given to proprioceptive inputs while younger children utilized visual information more. In a younger cohort, Bremner et al. (2013) tested reaching accuracy in 5–7-year-olds using a mirror illusion that placed proprioceptive and visual cues to arm location in conflict. The results showed evidence of visual capture of perceived hand location which increased up until 6 years of age.

In summary, although this body of research points to a maturation of sensory integration skills during childhood, the age at which children are reported to become adult-like in flexibly re-weighting sensory inputs appears to vary considerably. This could be due to the extent that the task relies on motor skills (i.e., pointing to the target/hand), temporal processing and/or working memory, all of which improve significantly over childhood (Takahashi et al., 2003; Gathercole et al., 2004; Barkley et al., 2014; Greenfield et al., 2015, 2017).

As previous studies have demonstrated (van Beers et al., 1996, van Beers et al., 1999; King et al., 2010), the relative weighting of visual and proprioceptive sensory information is best determined by the presentation of incongruent input. However, it should also be noted that overcoming experimentally induced visuo-proprioceptive conflict through sensory integration mechanisms is not an instantaneous process; integration mechanisms have been shown to be incomplete or less tightly constrained in children than in adults (Cowie et al., 2013; Greenfield et al., 2015). Nonetheless, research employing mediated reality methods, have been successful in demonstrating that seeing one’s hand in one location while feeling it in another will rapidly alter the perceived location of that hand (e.g., Newport and Preston, 2011; Preston and Newport, 2011; Greenfield et al., 2015; Bellan et al., 2015). The current study therefore investigated the development of optimal integration in children by characterizing the developmental trajectory of sensory weighting in a task that promoted the integration of visual and proprioceptive information concerning hand position. Unlike King et al. (2010), who used a localization task, with different targets for vision and proprioception (circle vs. own hand), here we employ a hand localization task in which a virtual image of the participant’s own hand serves as the incongruent visual “target” as well as the proprioceptive “target” using a mediated reality device called MIRAGE (Newport et al., 2010). Seeing the actual body is more analogous to real life and provides more salient information compared to a visual target that merely signals the position of the body, which may affect the extent to which visual information is weighted. Furthermore, so that a measure of purely visuo-proprioceptive integration could be obtained, without the confound of movement as in previous research, hand localization in the current study was measured using a perceptual judgment task rather than a pointing task. The task required children to locate their right index finger after being exposed to either congruent or incongruent visuo-proprioceptive information regarding hand position. Age-related differences in unimodal accuracy were assessed by asking children to estimate the location of their unseen hand after viewing congruent information. The same task was completed after presenting children with incongruent visual and proprioceptive information to measure the developmental trajectory of optimal sensory integration and to assess age related differences in the degree that one or other sense dominated. A similar paradigm used by Bellan et al. (2015) found that the presence of incongruent visual information significantly affects hand localization, with estimates biased toward the visual location of the hand. Overall, adults weighted visual and proprioceptive information at approximately 60 and 40%, respectively. Based on previous observations that suggest young children are more driven by visual information during visuo-proprioceptive conflict in hand localization tasks (King et al., 2010; Cowie et al., 2013), we hypothesized that the weighting of proprioceptive information under conditions of visuo-proprioceptive conflict would increase with age. Due to inconsistent methodology and findings in the literature, it is difficult to make predictions about the precise age children are able to integrate and flexibly reweight sensory information, however, most research has indicated that children under 10 years tend to favor one sensory modality, usually vision, more strongly.

## Materials and Methods

### Participants

Seventy-five children aged 4-11 years (*M* = 8.44, *SD* = 1.94, 43 females, 8 left-handed) participated as part of a Summer Scientist Week event held at The University of Nottingham for which children were invited to complete short experiments. Children came from mid-to high socioeconomic backgrounds. Parents of all children completed the Social Aptitudes Scale (SAS; Liddle et al., 2008), which measures social skills, and the Strengths and Weaknesses of ADHD symptoms and Normal behavior rating scale (SWAN; Swanson et al., 2012), which measures positive attention and impulse control. Ratings on the SAS and SWAN are made by parents based on how they think their child compares in relation to peers of the same age. On the SWAN a rating of 0 is exactly average while any rating above average gains a negative value and below average is given a positive value (SWAN; Swanson et al., 2012), On the SAS a validation study carried out by Liddle et al. (2008) with 7,977 participants yielded a mean score of 24.6 and similar distributions across different age ranges (5–8; 9–12; 13–16) each with a modal score of 20. The British Picture Vocabulary Scale III (BPVS III; Dunn and Dunn, 2009), was used to assess verbal mental age and administered to ensure none of the children had a developmental delay. Handedness was determined by the hand with which a child used for writing/drawing.

Data from 11 children were excluded: nine children did not keep their hands still during the task, one (aged 4 years) did not want to complete the task, and age data for one child was missing, leaving 64 children (40 females, 7 left-handed) who were included in the analysis (Table 1). The remaining participants included: 5 (aged 4–5 years); 12 (aged 6–7 years); 29 (aged 8–9 years); and 18 (aged 10–11 years). In this final sample, data were missing for three participants on the SAS, three on the BPVS and four on the SWAN. However, no children were reported to have a clinical diagnosis of a developmental disability. The parents of all children gave written informed consent prior to testing and ethical approval for the experiment was granted by the University of Nottingham, School of Psychology Ethics Committee and was conducted in accordance with the ethical standards of the Declaration of Helsinki.

**TABLE 1:** Descriptive statistics for the sample.

Statistic:	Mean	*SD*	Min	Max
Age in years	8.78	1.79	4.51	11.95
BPVS raw score	120.72	21.21	59	156
BPVS standardized score	105.05	11.4	72	131
Social Aptitudes Scale	25.31	6.19	6	39
SWAN	–21.64	9.68	−74	43
SWAN inattentive subscale	–6.22	9.09	−24	21
SWAN hyperactive subscale	–7.38	9.68	−27	15

*Age statistics are reported for the whole sample (N = 64). For the remaining measures statistics are reported for the number of participants it was available for. BVPS, British vocabulary picture scale; SAS, Social Aptitudes Scale; SWAN, Strengths and Weaknesses of ADHD Symptoms and Normal Behavior Scale. Higher SWAN scores indicate more inattention and hyperactivity.*

### Experimental Setup

**FIGURE 1:**
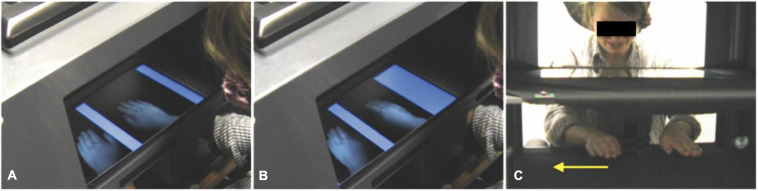
**(A)** At the start of the adaptation procedure, the seen location of the right hand matches its real location (note the alignment of the seen right hand and participant’s real arm). **(B)** Over the course of the adaptation procedure, the superimposed blue bars slowly expand to constrict the hand space. At the same time and without the participant’s awareness, the image of the right hand is shifted slowly leftwards so that in order to keep the hand visible between the blue bars, the participant must move their hand rightwards. This results in a separation between the seen and real location of the right hand (note the misalignment of the seen right hand and the participant’s real arm). In the actual experiment, a bib occluded the participant’s view of their arms. **(C)** The MIRAGE worksurface and participant’s hands from the experimenter’s viewpoint. The yellow arrow indicates the direction in which the right hand moves during the adaptation procedure. See electronic Supplementary Material 1 of Bellan et al. (2015) for a video of the MIRAGE adaptation procedure (incongruent condition).

Children knelt or sat on a chair to allow them to view their hands when placed on the work surface of the MIRAGE mediated reality device (Newport et al., 2010). The MIRAGE uses a rectangular horizontal mirror, suspended equidistant between the worksurface below and a computer screen above, to reflect live camera images of the hands displayed on the computer screen. These appear in the same physical location as the real hands with a minimal delay (∼16 ms) (see Figure 1), thus giving the child the impression that they were viewing their own hand, in its real location, in real time.

A black bib attached across the length of the mirror was tied comfortably around the participant’s shoulders to obscure a direct view of their upper arm. At the start of the task, a glove tip was placed on the child’s right index finger. This was referred to as “the finger with the hat on” so that there could be no confusion about which finger was being referred to during the experiment.

### Procedure

The basic task required children to make judgments about the location of their seen or unseen finger by verbally directing an arrow to be in line with their index finger after exposure to congruent or incongruent visuo-proprioceptive sensory input about the location of the hand. All participants were tested individually and took part in three conditions completed in the following order: congruent with vision of the hands (congruent seen; included to verify children understood the task and were competent in making verbal judgments of their hand position), congruent without vision (congruent unseen) and incongruent without vision (incongruent unseen). This particular order of conditions was important to ensure children were familiar with the MIRAGE system and understood how to judge the position of their hand before taking part in the more challenging incongruent condition.

In the two congruent conditions, the participant placed his or her hands on the worksurface of MIRAGE and watched as the experimenter moved their hands to a specified position. Both the left and right seen hands were in the same location as the real left and right hands, respectively. In the incongruent condition, before the experimenter placed the participant’s hands on the worksurface the individual took part in a visual adaptation procedure. The participant placed his or her hands in MIRAGE and held them approximately 5 cm above the workspace and were instructed to not touch blue bars which could be seen to box in each hand to the left and right (see Figure 1). The blue bars were graphically superimposed on the visual workspace and expanded slowly over the course of 25 s so as to constrict the space in which the hands could be positioned. During this period the spatial relationship between the seen location of the right hand and its real location was manipulated using an adaptation procedure modified from Newport and Gilpin (2011) and similar to that used in Bellan et al. (2015). This was achieved by moving the image of the right hand smoothly and incrementally leftwards at a rate of 4.5 mm/s. Thus, in order to keep the right hand in the same visual location the participant had to move their hand rightwards at the same rate with the result that after 25 s the seen hand was viewed 11.25 cm to the left of its true location. During the same period, the visual image of the left hand oscillated slowly leftwards and rightwards at an average velocity of 4.5 mm/s but ended up in the same location as it had started (i.e., with the seen left hand in the same location as the real left hand). This oscillation was included so that the movement of the image relative to the hand, and the tracking of that movement by the real hand, was equivalent across both hands. It is very rare for people to notice the movement of either hand relative to its seen image and conscious awareness of this has never been observed under experimental conditions (see Newport and Gilpin, 2011; Bellan et al., 2015). Once the adaptation procedure was complete, the participant’s hands were placed back down onto the worksurface of MIRAGE prior to them making judgments about the position of their right index finger.

After this initial period, the participant’s hands either remained visible (in the congruent seen condition) or were immediately occluded by replacing the visual scene with a blank image (in the congruent unseen and incongruent unseen conditions). Thus, the participant could either: see and feel the location of the hand simultaneously (congruent seen), only feel the location of the hand (congruent unseen) or feel the location of the hand having previously seen it in an incongruent location (incongruent unseen). The participant then estimated the location of the right index finger using the following procedure. For location judgments, the participants saw a red arrow (reflected from the computer screen above) traveling laterally across the MIRAGE workspace where his or her hands were located and said “Stop” when they thought that the arrow was directly in line with the finger wearing the hat (the right index finger). This would prompt the experimenter to immediately release a button on the computer keyboard immediately stopping the arrow from moving. The position of the arrow was then recorded in pixels along the *x*-axis. Each measurement was taken twice for each condition, once with the arrow traveling from right to left and once from left to right (order counterbalanced across conditions and participants). In all conditions, the hands were resting on the worksurface of the MIRAGE throughout the duration of the judgment task. The total duration of the experiment, including set-up and explanation of the task, was approximately 10 min.

### Statistical Analysis

Localization error scores were calculated for each participant for each of the three conditions in the following way. For each trial the *x*-axis co-ordinate of the position of the tip of the right index finger was recorded in pixels (100 units equates to 7.5 cm). For each condition, the average of the two estimates of finger position was calculated and subtracted from the actual finger position to give an estimate of localization error. A score of zero would represent a completely accurate estimate of hand location. Positive values indicated estimates to the right of the actual finger location and negative values indicated estimates to the left (i.e., closer to the midline). In the incongruent unseen condition, the hand was seen 11.25 cm to the left of the real location; thus, a score of zero in this condition would represent total reliance on proprioception, a score of −11.25 would represent total reliance on vision. Scores in between these values indicate the level of weighting given to proprioception and vision, respectively, with −5.625 having equal weighting.

A developmental trajectory analysis was conducted to address the main research questions which involved two steps. Firstly, the within-subjects effect of condition on localization error was explored using a one-way repeated measures ANOVA. This allowed us to directly investigate the influence of incongruent visual information on proprioception in comparison to conditions when visual and proprioceptive information are congruent. Next to assess developmental change in localization error and importantly how it interacts with performance on the different conditions the analysis was re-run as an ANCOVA with rescaled age entered as a covariate in accordance with a developmental trajectory approach (Thomas et al., 2009). Investigating the main effect of condition separately from the condition by age interaction is recommended (Thomas et al., 2009) because the addition of a covariate changes the main effect of the within-subjects factor leading to an overly conservative estimate of the effect (Delaney and Maxwell, 1981).

In addition to our main analyses, further regressions were carried out to explore secondary questions in regards to other factors that might influence performance based on previous research. As previous research (King et al., 2010) found a positive relationship between proprioceptive accuracy and weighting of proprioceptive inputs over and above the effect of age a regression analysis was conducted. This analysis was only carried out on the congruent unseen condition which gave an estimate of baseline proprioceptive accuracy and the incongruent unseen error which measured proprioceptive weighting. Specifically, a hierarchical regression model was used to control for age effects on performance by entering it at the first step so the relationship between proprioceptive accuracy and proprioceptive weighting could be explored independently. A second hierarchical regression was also conducted to explore whether general attentional skills (as measured by the SWAN) and social skills (as measures by the SAS) influenced localization accuracy on the incongruent unseen condition. Age and congruent unseen scores were entered at the first step, with SWAN and SAS scores entered at the next step.

## Results

**FIGURE 2:**
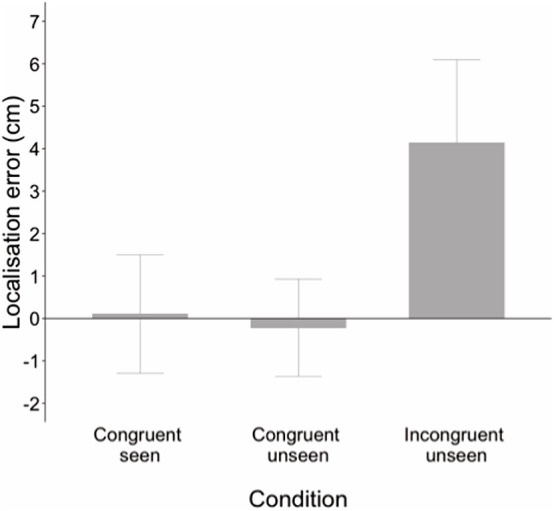
Localization error in cm for each condition across the whole sample. Positive values represent mislocalization to the left of the real hand; negative values represent error to the right of the real hand. Error is low in both congruent conditions, but significantly increased when visual and proprioceptive inputs were incongruent. Error bars ± 1 SD.

Figure 2 shows performance in each condition across the whole sample. The one-way repeated measures ANOVA revealed a main effect of condition on localization error, *F*(1, 63) = 151.70, *p* < 0.001, η*_*p*_*^2^ = 0.716. Pairwise comparisons (Sidak adjustment for multiple comparisons) revealed no significant difference in accuracy between the congruent seen and congruent unseen conditions (*p* = 0.159) but significant differences were found when incongruent unseen was compared to the congruent seen and congruent unseen conditions (both *p* < 0.001). Children were highly accurate at locating their index finger when congruent visual and proprioceptive information was available, indicating that they all understood the task. Accuracy remained high in the congruent unseen condition, when only proprioceptive inputs were present at judgment. However, as predicted, accuracy was significantly reduced in the incongruent condition compared to both congruent conditions.

**FIGURE 3:**
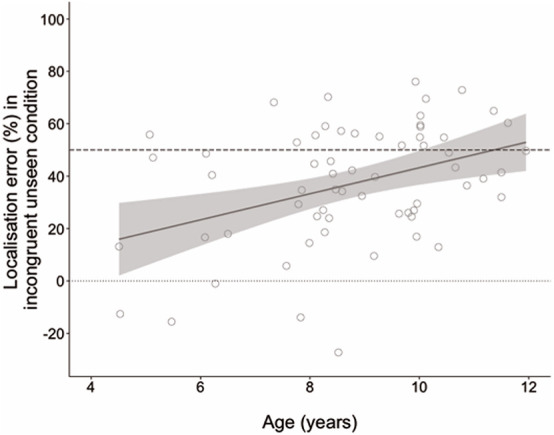
Localization error as a percentage of distance between seen and actual hand locations in the incongruent unseen condition in which the seen and real hands were in different locations. The dashed line at 50% indicates equal weighting of vision and proprioception; the dotted line at zero indicates complete reliance on proprioception. Negative values indicate estimates beyond the real hand location. Shaded region shows 95% confidence interval.

An ANCOVA was performed entering age as a covariate to compare developmental change in localization error between conditions. This analysis revealed a main effect of age, *F*(1, 62) = 7.64, *p* = 0.007, η*_*p*_*^2^ = 0.110, but also a significant condition by age interaction, *F*(1, 62) = 12.77, *p* = 0.001, η*_*p*_*^2^ = 0.171. Parameter estimates showed that age did not predict performance in the congruent seen, *B* = −0.004, *t*(62) = 1.64, *p* = 0.106, or congruent unseen conditions, *B* = −0.008, *t(*62) = −1.11, *p* = 0.272. However, age was a significant predictor of performance in the incongruent unseen condition, *B* = −0.046, *t*(62) = −3.34, *p* = 0.001. As age increased, localization estimates were increasingly further from the actual hand and closer to the seen hand. Age explained 15% of the variance in accuracy scores in the incongruent unseen condition (*R*^2^ = 0.153). Figure 3 displays the developmental trajectory for this condition, with localization error converted into a percentage of the distance between the seen and actual hand locations to demonstrate how the weighting of vision and proprioception changed with age.

### Regression Analyses

A hierarchical regression was conducted with age (in months) entered at the first step and congruent unseen error (absolute value) entered next as a predictor with incongruent unseen error (i.e., percentage of distance between seen and unseen hand) as the outcome variable. Congruent unseen error was not a significant predictor of accuracy in the incongruent unseen condition, *B* = −5.63, *t*(62) = −1.54, *p* = 0.129.

To investigate whether general attentional or social skills predicted accuracy (i.e., error as distance percentage) of estimates in the incongruent unseen condition, age and congruent unseen accuracy scores were added as predictors into the first block of a hierarchical regression model, with SAS and SWAN inattentive subscale scores entered in the second block. Seven participants (10.94%) were excluded from the regression due to list-wise missing data across measures. Neither SAS [*B* = 0.48, *t*(59) = 0.84, *p* = 0.40] or SWAN [*B* = 0.13 *t*(59) = 0.93, *p* = 0.36] scores predicted localization error on the incongruent unseen condition.

## Discussion

The present study investigated the relative contributions of visual and proprioceptive inputs on the development of body localization in primary school-aged children. When given incongruent visual and proprioceptive information about the location of the hand, younger children (<10 years) favored proprioceptive input more than older children who weighted vision and proprioception more equally. The developmental trajectory for multisensory integration in this task was not affected by variability in social skills or inattention.

As expected, all children were highly accurate in locating the finger in the congruent seen condition (see Figure 2), indicating that they understood the task and could easily indicate the location of their seen hand by 4 years of age. Children’s estimates were also accurate in the congruent unseen condition, when congruent vision of the hand had been removed and only proprioceptive information was available. Again, performance did not improve with age suggesting that younger children are equally good at using proprioceptive information as older children to localize the hand when this is not aided by visual inputs. One might argue that in the congruent unseen condition visual information about the location of the target had recently been available so it is possible that children could have used a memorial representation, or visual trace, of the hand’s visual location in this condition. However, if this were the case then we would have also found visual anchoring in performance on the incongruent unseen condition, but instead location estimates were in between the seen and real location of the hand. Furthermore, estimates for younger children were shifted more toward the proprioceptive (true) location. Younger children appeared to rely more on proprioception to locate their unseen finger while older children weighted visual inputs more strongly. The nature of this sensory integration was not related to proprioceptive accuracy in the congruent unseen condition and did not appear to be influenced by variability in social aptitude or inattention.

It is interesting that these results appear to contradict previous research that observed greater weighting of visual over proprioceptive information in early childhood. For instance, in the hand localization task conducted by King et al. (2010) it was found that older children upweighted proprioceptive information (i.e., actual finger location) more than younger children. Although the discrepancy between visual and proprioceptive information was smaller in King et al. (2010), the abrupt onset of the incongruent visual indicator (i.e., a target circle) in a different location than the proprioceptive target (i.e., unseen finger) may have made the disparity more salient. Thus, older children may have actively discounted the visual information and instead favored the more reliable proprioceptive information. In the current study, by contrast, the separation of visual and proprioceptive information was gradual and constant during the adaptation process allowing hand location to be recalibrated without reaching conscious awareness. Secondly, the nature of the visual information in the current study, being a live image of the participant’s own hand, was much more likely to be embodied as pertaining to the body than a target circle representing finger location in King et al.’s (2010) study. In everyday life, visual cues of limb localization originate from vision (and proprioception) of the body rather than from visual targets signaling body position. This argument is supported by research which has shown body ownership of a virtual hand is stronger for images that look more like one’s actual hand (Ratcliffe and Newport, 2017; Pyasik et al., 2020). Thus, the current experimental conditions were perhaps more likely to induce sensory integration of signals related to the body due to the use of a virtual image of the participant’s own hand.

Nonetheless, the current results also contrast with other research findings where an image of a hand was used as the visual representation. A stronger reliance on vision in younger children was observed by Bremner et al. (2013) in a task requiring a visually driven response under conditions of visuo-proprioceptive conflict. Visual and proprioceptive information about the limb were placed in conflict by reflecting the left hand in a mirror located asymmetrically between the hands so that it appeared (visually) to be the right hand but was not in the same physical location as the real right hand (which was hidden behind the mirror). The task involved pointing to a visual target with the unseen hand while the reflected left hand was in view and, presumably, perceived to be the right hand due to the nature of the illusion. Vision dominated (or captured) subsequent processing of limb position with children tending to point from the seen position of the hand rather than the felt position. Since the task necessitated visually guided reaches with the seen (albeit incorrectly positioned) hand to a visual target, this was a primarily visual-driven task and, as such, vision might be expected to dominate. The current task conducted in MIRAGE by comparison was primarily proprioceptive in nature (verbally guiding an arrow to the felt location of the unseen hand). If vision and proprioception are not integrated effectively at a young age, but instead are either processed independently or are treated such that one sense is strongly dominant over the other, then a task which favors the processing of proprioceptive inputs might produce outcomes with a strong proprioceptive bias. Under this hypothesis, children are still integrating information probabilistically, as suggested by King et al. (2010), but the weighting of sensory information is heavily influenced by the development of multisensory integration abilities rather than (or as well as) the development of unisensory capabilities. Importantly, an immature development of this integration process, could lead to a bias in processing either visual, proprioception, or another sensory input depending on which is the most salient in a given task.

In a previous study using a similar task in adults, Bellan et al. (2015) found that localization errors in the incongruent condition were consistent with a bias toward visual information, which was given a weighting of approximately 60%. In the current study, the performance of the older children was approaching this adult benchmark, with 10–11-year-olds (*n* = 18) judging the real hand to be ∼50% of the distance to the seen hand. By contrast the youngest children, 4–6-year-olds (*n* = 11), judged the distance at less than 30% toward the seen hand. We contend, therefore, that the results of this experiment demonstrate that visuo-proprioceptive integration develops throughout childhood from very little integration at 4 years to almost adult-like at 11.

In the current study, the three conditions were presented to participants in a fixed order—congruent seen, congruent unseen and incongruent unseen. This was done to ensure that the children understood the task and were able to complete the non-illusory conditions first before completing the critical illusory trials (incongruent unseen). It is important to note that children were not given any feedback about their accuracy so as not to influence their performance in the subsequent conditions. The duration of the experiment was relatively short, taking a total of less than 10 min. Therefore, it is unlikely the age-related differences observed in the incongruent unseen condition are due to fatigue; if this were the case, we would expect the performance of younger children to be random. However, the results indicate a systematic difference in the way in which younger children integrate visual and proprioceptive inputs, with a clear developmental trend in performance on this task.

The experiment only measured localization of the right hand, which was the dominant hand for the majority of children in this sample. In future work, it might be interesting to investigate whether similar effects are observed for localization of the non-dominant hand. Studies have found an attentional bias for the dominant side of space (Rubichi and Nicoletti, 2006), which could have an effect on the extent to which visual information is prioritized during integration during body localization.

In summary, developmental trajectory analysis of a hand localization task in primary school age children suggests that while localization of the seen and unseen hand in children is consistently good, when visual and proprioceptive input are incongruent, localization estimates reveal differences in the integration of multisensory information related to the body which younger children appear to integrate less optimally than older children.

## Data Availability Statement

The raw data supporting the conclusions of this article will be made available by the authors, without undue reservation.

## Ethics Statement

The studies involving human participants were reviewed and approved by the School of Psychology Ethics Committee at the University of Nottingham. Written informed consent to participate in this study was provided by the participants’ legal guardian/next of kin. Written informed consent was obtained from the individual(s) for the publication of any potentially identifiable images or data included in this article.

## Author Contributions

NR, KG, DR, and RN developed and planned the study. NR, KG, and EH collected data. NR and KG analyzed the data and wrote the first draft of the manuscript. NR, KG, EH, DR, and RN edited and developed the manuscript. All authors contributed to the article and approved the submitted version.

## Conflict of Interest

The authors declare that the research was conducted in the absence of any commercial or financial relationships that could be construed as a potential conflict of interest.

## Publisher’s Note

All claims expressed in this article are solely those of the authors and do not necessarily represent those of their affiliated organizations, or those of the publisher, the editors and the reviewers. Any product that may be evaluated in this article, or claim that may be made by its manufacturer, is not guaranteed or endorsed by the publisher.
